# Current Understanding of the Pathogenesis of Porcine Circovirus 3

**DOI:** 10.3390/pathogens11010064

**Published:** 2022-01-04

**Authors:** Chaitawat Sirisereewan, Roongroje Thanawongnuwech, Roongtham Kedkovid

**Affiliations:** 1 Department of Veterinary Pathology, Faculty of Veterinary Science, Chulalongkorn University, Bangkok 10330, Thailand; cs.chaitawat@gmail.com; 2 Department of Veterinary Medicine, Faculty of Veterinary Science, Chulalongkorn University, Bangkok 10330, Thailand; 3 Swine Reproduction Research Unit, Chulalongkorn University, Bangkok 10330, Thailand

**Keywords:** circovirus, emerging, pathogenesis, pig, porcine circovirus 3

## Abstract

Circoviruses are closed, circular, single-stranded DNA viruses belonging to the family *Circoviridae* and the genus *Circovirus*. To date, at least four porcine circoviruses (PCVs) have been recognized, including PCV1 to PCV4, respectively. Similar to PCV2 pathogenesis, PCV3 has been reported worldwide with myriad clinical and pathological presentations such as reproductive disorders, respiratory diseases, diarrhea etc. Current understanding of PCV3 pathogenesis is very limited since the majority of studies were mostly field observations. Interpretation of the results from such studies is not always simple. Various confounding factors affect the clinical appearance and pathological changes of the infected pigs. Recently, several experimental PCV3 infection studies have been reported, providing a better understanding of its pathogenesis. In this review, we focused on novel findings regarding PCV3 pathogenesis from both field observation and experimental infection studies. Possible factors involved in the conflicting results among the experimental infection studies are also discussed. This review article provides important insight into the current knowledge on PCV3 pathogenesis which would aid in prioritizing research in order to fill the knowledge gaps.

## 1. Introduction

Circoviruses are one of the six families of circular Rep-encoding ssDNA (CRESS DNA) viruses, belonging to the family *Circoviridae* and the genus *Circovirus*. To date, at least four porcine circoviruses (PCVs) have been recognized, including PCV1 to PCV4, respectively. PCV1 was first detected in a porcine kidney cell line (PK-15) and is generally considered non-pathogenic. A spotlight has been shone on PCV2 since 1995 when the virus was suspected as the causative agent of a disease called ‘postweaning multi-systemic wasting syndrome’ (PMWS) in Canada [1,2]. PCV2 infection has been described with various clinical manifestations, called porcine circovirus associated disease (PCVAD), including reproductive failure [3], porcine dermatitis and nephropathy syndrome (PDNS) [4], and respiratory diseases [5]. While the swine industries were struggling with PCV2, PCV3 and PCV4 were discovered [6,7,8]. PCV3 can be found in pigs, showing varied clinical signs, and the virus has been detected in many countries worldwide. Currently, PCV4 has been reported in China [8] and Korea [9], with limited data on the fundamentals of its pathogenesis and economic impact.

PCV3 was first identified in 2015; however, it was not until recently that the pathogenesis started to be elucidated. The virus was discovered by metagenomic sequencing from an outbreak in sows which exhibited PDNS-like lesions and increased aborted and mummified fetuses. Since the discovery of PCV3, the virus has been reported in domestic pigs worldwide with different clinical outcomes. The myriad clinical and pathological presentations associated with PCV3 infection had been suggested to include reproductive disorders, PDNS [7], systemic inflammatory disease [6], respiratory disorders [10,11,12], diarrhea [10,13], and central nervous system signs [14,15]. In those studies, the virus was also detected in apparently healthy pigs, therefore some unknown factors may be involved in PCV3 disease development and its pathogenesis. Virus isolation has long been the major obstacle in studying PCV3 pathogenesis since isolation and propagation in continuous cell lines have been mostly unsuccessful. However, PCV3 has recently been isolated using porcine kidney-15 cell line (PK-15) and primary porcine kidney cells [16,17]. Moreover, a PCV3 infectious clone was successfully constructed [18]. With these breakthroughs, appropriate pathogenesis studies using experimental infection are possible.

This review article summarizes what has been found from experimental infection studies and field observations, and could be helpful in understanding PCV3 pathogenesis. Possible mechanisms underlying certain PCV3 diseases are also discussed.

## 2. Pathogenesis of Certain PCV3 Associated Diseases

**Figure 1:**
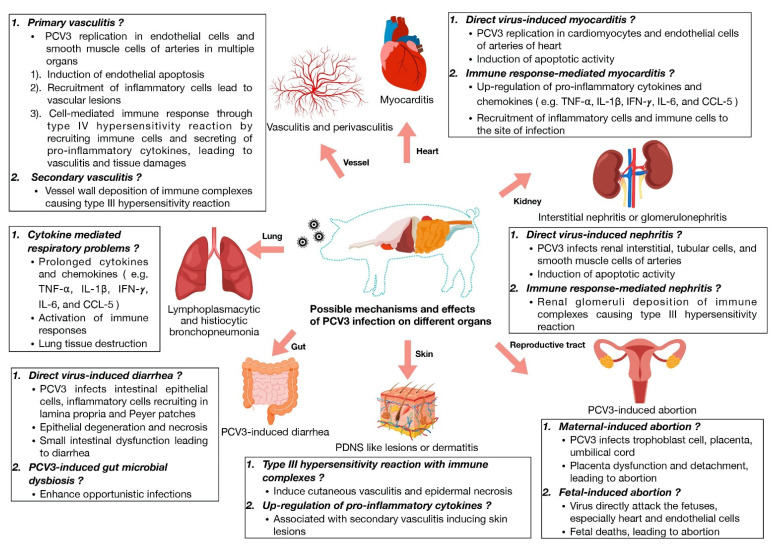
Possible mechanisms of PCV3-induced pathology.

Virus infection can induce inflammation and tissue injury via various mechanisms. At least three major categories are recognized, including (1) virus-induced, e.g., virus-induced apoptosis; (2) immune-response-mediated, e.g., immune system responding to eliminate infected cells; and (3) immune-complex-mediated mechanisms, e.g., type III hypersensitivity. Inflammation and tissue injury following PCV3 infection might be due to these mechanisms (Figure 1). From both experimental and natural PCV3 infection, virus replication and inflammation frequently coincided in various tissues [15,17,18,19]. Virus-induced and immune-response-mediated inflammation and/or tissue injury could be suggested in these scenarios, however the absence of virus inside the lesions was also observed. Thus, immune-complex-mediated mechanisms, among others, might also be speculated. It should be noted that the absence of PCV3 or PCV3 replication in this scenario could be due to levels being below the level of detection of the method utilized [19]. Moreover, studies identifying immune complex accumulation in the lesions of PCV3 infected pigs are still lacking. Further investigations are needed to clarify whether or not immune-complex-mediated inflammation is involved in PCV3 disease.

### 2.1. Myocarditis, Nephritis and Vasculitis

Myocarditis, nephritis and vasculitis were among the common lesions found in several reports, including field observation and experimental infection studies. Moreover, these lesions could be found in subclinically infected pigs. Therefore, it is interesting to consider whether these lesions might be fundamental in PCV3-induced disease; or how the disease could shift from subclinical myocarditis, nephritis and vasculitis to a more severe form, such as PDNS-like disease, in certain scenarios. 

Induction of myocarditis, nephritis and vasculitis possibly occur via virus-induced or immune-response-mediated mechanisms, since PCV3 presence and/or replication has been observed in these tissues [6,7,15,17,18,19,20]. Microscopic evaluation indicates apoptotic activity associated with pyknotic nuclear debris found in the cardiac lesions [6,18]. In the kidney, PCV3 antigen or PCV3 replication was found in the renal interstitium, tubular epithelial cells, endothelial cells, the smooth muscle of renal arteries, and infiltrating inflammatory cells with the presence of interstitial glomerulonephritis and/or interstitial nephritis [15,17,18]. Vasculitis in PCV3 infected pigs ranged from local to systemic perivasculitis [6,7,15,17,18,20]. Importantly, vasculitis was found in the cardiac, renal and intestinal tissues in experimentally infected pigs [17,19]. Reactome pathway analysis has shown that following PCV3 infection, proteins associated with adhesion and cell junction organization were expressed [21]. In PCV2, intercellular adhesion molecule 1 (ICAM-1) was upregulated in the infected endothelial cells, which might modulate the leucocyte infiltration into tissues leading to vascular lesions [22]. Whether ICAM-1 might play an important role in PCV3-induced vascular damage should be further investigated. Moreover, upregulation of pro-inflammatory cytokines (e.g., TNF-α, IL-1β, IL-6, and IFN-γ) in sera was also observed in PCV3-infected pigs [18]. Therefore, host immune responses against PCV3 infection might be responsible for the observed lesions as well. 

Together with inflammation, PCV3-induced apoptosis might also be central to the injury of cardiac, renal and vascular tissues in the infected pigs. ORF3 protein of the *Circoviridae* family could also induce apoptosis [23], however, functions of PCV3 ORF3 protein have not been fully characterized.

Immune complex-induced glomerulonephritis has been reported in other swine viral infections including classical swine fever virus (CSFV) [24] and PCV2 [25]. Detection of immune complexes in renal glomeruli for either CSFV or PCV2 infected pigs revealed accumulation of IgG, IgM, and complement factors (C1q or C3), but absence of viral antigens. However, immune complex deposition in the renal glomeruli of PCV3-infected pigs has not been established.

### 2.2. Porcine Dermatitis and Nephropathy Syndrome (PDNS)

Porcine dermatitis and nephropathy syndrome (PDNS), caused by type III hypersensitivity reaction, is defined by systemic vasculitis with marked tropism for the skin and kidney [26]. Systemic vasculitis with dermal and epidermal necrosis and necrotizing and fibrinous glomerulonephritis are hallmark lesions of PDNS [25,27]. Currently, PDNS-like disease might arguably be the most severe disease form following PCV3 infection in both natural and experimental infections [18,20,21]. However, the disease development was not clearly elucidated. 

In PCV2 pathogenesis, immune complexes composed of accumulating IgM, IgG, and complement factors C1q and C3 were found in renal glomeruli of PCV2-infected pigs [25]. Moreover, infected pigs with PDNS also had upregulated pro-inflammatory cytokines (e.g., IL-1α, IL-2, IL-4, IL-6, IL-12, TNF-α and IFN-γ) [28]. Currently, although PCV3 has been found in pigs showing PDNS-like lesions in field observations (ref), only one experimental PCV3 infection study could induce such lesions [18]. In that study, upregulation of pro-inflammatory cytokines and chemokines, such as TNF-α, IL-1β, IFN-γ, IL-6, and CCL-5, could be seen [18]. However, immune complex accumulation was not measured in that study. In contrast, a different experimental PCV3 infection study resulted in subclinical infection in which PCV3-infected pigs showed lower or average baseline pro-inflammatory cytokines [19]. Abundant eosinophil infiltration was found in tracheobronchial, mesenteric, and inguinal lymph nodes of pigs showing PDNS-like diseases following experimental PCV3 infection [18]. The role of eosinophils in PCV3 diseases is not yet known. Eosinophils can function as an antigen-presenting cells (APC) in response to viral antigen stimulation and secrete cytokines associated with T-cell responses [29,30,31]. Eosinophil-related antiviral mechanisms can also be found against other respiratory viruses, including respiratory syncytial virus and influenza [32]. Further studies are needed to clarify the association between PCV3 and PDNS.

### 2.3. Reproductive Failure

Although PCV3-induced reproductive failure was not studied in the experimentally infected pigs, various field observations suggest that the virus might be the cause. Currently, fetal infection was thought to be the major mechanism underlying PCV3-induced reproductive failure.

PCV3 antigens were found in pig farms experiencing reproductive failure with high viral titers [10,33]. Moreover, vertical transmission to fetuses can be observed as evidenced by high viral titers and pathological changes in multiple organs of aborted and stillborn fetuses [20,34]. Causes of reproductive disorders can be divided into maternal and fetal-induced abortion. High PCV3 titers in aborted fetuses [20,35] and PCV3 replication observed in the fetal heart, placenta, and endothelial cells could be confirmed by in situ hybridization (ISH) [15], which likely indicated that PCV3 could directly affect the fetuses. The variable sizes of crown-to-rump length also be observed in mummified fetuses [35] suggests inter-fetal transmission. Multiplication of PCV3 in fetal heart, placenta, and endothelial cells might be responsible for viral dissemination to other organs leading to fetal microscopic lesions and fetal death. Observed microscopic findings were mild to moderate lymphoplasmacytic vasculitis in various tissues, myocarditis, and endocarditis [15,20,35,36]. Similarly, high PCV2 virus load in fetal tissues associated with myocarditis were also found in PCV2-induced abortion causing fetal deaths [37]. High PCV3 virus load was also found in the thymus and lymph nodes of aborted and weak-born piglets, suggesting additional tissue tropism of PCV3 in fetuses [34]. Interestingly, PCV3 genetic materials could be detected in the affected tissues from mummified fetuses and stillborn piglets without obvious microscopic lesions [38] and gross lesions [34]. Recently, PCV3 viral load has been found in the whole reproductive tract including ovary, oviducts, uterine horns, and ovarian lymph node of sows aborting at the early stage of gestation (Sirisereewan et al., unpublished data). PCV3 could also be found in trophoblast cells, placenta, and umbilical cord from mummified or stillborn fetuses [15,34,39,40]. Interestingly, it has been demonstrated that PCV2-infected fetuses have focal necrosis of chorionic epithelium, especially the trophoblasts [41]. Thus, viral-infected trophoblasts might play a major role in placental dysfunction, leading to complications including placental tissue layers’ integrity resulting in placental detachment and fetal death. The mechanism of reproductive failure induced by PCV3 remains unclear and needs to be further investigated. Farms experiencing reproductive problems in the presence of PCV3 should be investigated and interpreted carefully. 

### 2.4. Respiratory Disease and Diarrhea

Respiratory diseases and diarrhea can be found in naturally infected pigs [10,11,42], though those might not be considered as the major clinical signs following PCV3 infection and various factors could contribute to the clinical signs. Therefore, the ability of PCV3 to induce respiratory disease and diarrhea is still questionable. Since such clinical signs are common in swine production, the impact of PCV3 should be thoroughly investigated.

The reproduction of respiratory signs in experimentally infected pigs is currently controversial. In one study, the infected pigs showed various clinical presentations including respiratory signs such as coughing, sneezing, and respiratory distress with the presence of PCV3 antigen in the lung tissues [18]. The authors noted that, in those pigs, lesions were not limited to the respiratory tract. Severe PDNS-like lesions were observed, affecting various tissues. Histopathological lesions showed lymphoplasmacytic and histiocytic bronchointersitial pneumonia. Neutrophil recruitment was also observed [18]. However, other studies have failed to induce pathological changes in the lung and were unable to demonstrate PCV3 replication in the lung tissue [17,19]. In fact, the pigs only showed subclinical infection [17,19]. The absence of lung lesions in the later studies might not be unexpected since the virus was not found in the lung. At the moment, the factors involving lung tropism of PCV3 and the pathogenesis of the respiratory disease are not yet known. The causes of the controversial results from those studies are discussed later in this review.

Experimental infection studies also showed conflicting results regarding induction of diarrhea and pathological changes in the small intestine. One study found that pigs challenged with PCV3-positive intestinal contents (derived from diarrhea from pigs in which several pathogens were ruled out from the inocula) developed diarrhea with moderate to severe villous atrophy associated with small intestinal epithelial degeneration and necrosis [13]. In a study resulting in PDNS-like lesions [18], the infected pigs also developed diarrhea showing small intestinal epithelial cell degeneration and necrosis. PCV3 antigen was found in the intestinal epithelial cells, inflammatory cells in lamina propria, and Peyer’s patches. In the studies showing subclinical infection [17,19], although neither diarrhea nor intestinal epithelial degeneration were observed, lymphoplasmacytic periarteritis was found in the intestine. The dynamic changes of the microbiota induced by PCV3 infection were also reported [43], and gut microbial dysbiosis might lead to an increased number of pigs exhibiting diarrhea. It might be speculated that PCV3 plays a role in gastrointestinal associated disease, however, the pathogenesis needs further investigation.

## 3. What Have We Learnt from the Recent Experimental Infection Studies?

Field observation studies provide valuable information regarding PCV3 pathology. However, interpretation of the results from such studies is not always simple. Various confounding factors can affect the clinical appearance of the infected pigs. On the other hand, virus inoculation studies in the controlled environment are more straightforward but might not accurately reflect the field environment.

At least six experimental PCV3 infection studies have been reported to date [13,17,18,19,21,43]. Four of those studies investigated the clinical presentation of infected pigs, referred to as Study A [18], B [17], C [19] and D [13], respectively in this section. Studies A–C examined overall disease occurrence including clinical signs, pathological changes, virus replication, and immune responses. Study D exclusively explored diarrhea induced by the virus. The remaining two of the six studies investigated gut microbiota [43] and lung proteome profiles [21] of PCV3-infected pigs. Those two studies also used the same virus strain as in Study A (PCV3/CN/Hebei-LY/2015). 

Although Studies A–D explored PCV3 pathogenesis using experimental infection, details of the study methods varied to a certain extent. Selected parameters of the study methods are shown in Table 1. In those studies, clinical signs were first observed in pig farms (farms of origin) and PCV3 was then identified. PCV3 from those farms were later used as the inocula in the experimental infection studies. In the experiments, pigs received the virus alone or together with immunostimulant and various parameters were monitored after inoculation, such as clinical signs, macroscopic and microscopic lesions, PCV3 titers and/or replication in various tissues, and PCV3-specific antibody responses. Results and implications of these parameters are discussed later in this review. 

Drawing a conclusion on PCV3 pathogenesis from those studies is challenging since the results varied. To illustrate the differences of the overall results, five parameters were selected and scored among the studies, i.e., the value was the highest, the middle or the lowest of the three studies (Table 2). Study D focused only on diarrhea and is not included in Table 2 since some data were not available. Briefly, Study A showed highest disease severity with clinical diseases and mortality, while Studies B and C showed only subclinical infection.

### 3.1. Clinical Signs and Pathological Changes from the Experimental Infection Studies

Studies A and D successfully produced clinical diseases. In Study A, various clinical signs were observed such as fever, reddening of skin and ears, papules, coughing, sneezing, diarrhea, and convulsions, suggesting systemic disease. In Study D, infected pigs showed diarrhea. Clinical signs were not observed in either Study B or C. 

In Studies A and D, all inoculated animals showed clinical signs (5/5 and 6/6 pigs, respectively). Two of five pigs died in Study A. This was in stark contrast with Studies B and C, where none of the inoculated pigs showed clinical signs (0/8 and 0/6 pigs, respectively). The absence of clinical signs might be caused by various factors. For example, the virus strains used might be of lower pathogenicity. This should be further investigated since the association between PCV3 genetics and disease outcomes is currently unknown and the duration of the studies might not have been sufficient. Studies A–D ended at seven to 42 days post inoculation (dpi) (Table 1). At the end of the studies, all inoculated pigs still showed viremia (except for Study D, in which viremia was not measured). Moreover, at the end of Studies B and C, PCV3 replication was still found in various tissues indicating ongoing virus infection [17,19]. Therefore, it is possible that disease might occur later in infection (e.g., after 42 dpi), as has already been suggested [19]. The impact of this chronic infection should be studied.

Pathological changes were different among Studies A–D. Study A showed the highest disease severity with PDNS-like disease presentation, while Studies B and C only induced subclinical infection. Study D only showed diarrhea. Gross and histopathological lesions were studied only at the end of each study (Table 1), except for Study C (discussed later). Gross lesions were observed only in Studies A (PDNS-like disease) and D (diarrhea). In Study A, lesions were found in various tissues, such as lobular pneumonia with mottled tan consolidation, enlarged lymph nodes, swollen kidneys with hemorrhagic foci, and swollen spleen. However, microscopic lesions were found in all studies (Table 3). In Studies A–C, lymphocyte infiltration was found in various tissues. Lymphoplasmacytic bronchointersitial pneumonia found in Study A was related with the respiratory signs. It should be noted that degeneration of epithelial cells of the small intestine might explain diarrhea observed in both Studies A and D. Despite the differences in disease severity observed in those studies, myocarditis and nephritis were the common lesions found in Studies A–C. 

Histopathological changes associated with IHC or ISH presence of PCV3 were found in multiple tissues (Table 3). Influence of PCV3 tissue tropism and disease induction should be clarified. Lymphocyte necrosis and lymphoid depletion were also found in various lymphoid tissues in Study A (Table 3). Whether the lymphoid involvement is a key determinant of PCV3 disease severity should be further studied. The pathological changes studied in these works were limited to certain time points, therefore pathological findings during the chronic stage of infection after 28–42 dpi should be further investigated. In Study C (subclinical infection), histopathology was studied at 21 and 48 dpi, but lesions were observed only at 48 dpi. It showed that histopathological changes started between 21–42 dpi. Therefore, it might not be unexpected that macroscopic changes and clinical signs (if any) were not observed prior to 48 dpi. In other words, it could imply that the progression of certain forms of PCV3 disease can be somewhat slow [19], which was in contrast with Studies A and D.

### 3.2. PCV3 Infection Kinetics and Dynamics in the Experimental Infection Studies

Primary replication sites of PCV3 were not determined in these studies. Virus distribution or replication was studied at 28 dpi for Studies A and B, and at 42 dpi for Study C; virus distribution or replication was not measured in Study D. Therefore, information regarding PCV3 infection kinetics was very limited. 

The heart and kidney seemed to be important sites for PCV3 replication. Virus replication sites and tissue tropism based on histopathological lesions are shown in Table 3. In Study A, immunohistochemistry (IHC) was used to determined virus distribution in the infected pigs. Studies B and C used in situ hybridization (ISH) to identify virus replication in tissues. Study B showed the narrowest tissue tropism while Study A had the widest tissue tropism. However, since Study A did not directly measure virus replication in the tissues, the results might not be comparable. It is interesting that virus antigen or virus replication was identified in the heart and kidney in Studies A–C, as well as the results from the field observation [15]. 

Tissue tropism and virus titer might be associated with disease virulence, at least in certain scenarios. Study A showed the widest tissue tropism and most severe clinical outcomes. Compared with Studies B and C (subclinical infection), PCV3 was also found in lung and lymph nodes in Study A (Table 3). The role of tissue tropism and the impact of PCV3 replication in lung and lymph nodes should be further investigated. In addition to the widest tissue tropism, Study A also showed the highest peak viremic titers. After the PCV3 inoculation in all studies, viremia was identified in all pigs except for pigs of Study D. In Study A (PDNS-like disease), PCV3 titers were already high in the sera at 7 dpi (6–7 log10 copies/ml) and the titers peaked at 8.89 log10 copies/ml at around 21 dpi. This viremia level could be considered high when compared with PCV3 titers from previous studies, which generally did not exceed 8 log10 copies/ml [11,44,45]. In Study B (subclinical infection), PCV3 titers started to increase during 7–14 dpi. The titers peaked at 6.09 log10 copies/ml around 21–28 dpi (the end of the study). Peak viremia titers were not available for Study C (subclinical infection) where the data are only shown in graph without data labels; approximation indicates it would be between 7–8 log10 copies/ml. It has been shown in PCV2 pathogenesis that the level of viremia might be associated with PCVAD [46]. This should be further studied in PCV3 pathogenesis and its tissue tropism. 

As previously mentioned, viremia was still observed at the end of Studies A–C (28–42 dpi), with the titers approximately between 6.0–7.5 log10 copies/ml. For PCV2 infection, viremia could still be observed at 140 dpi after a single PCV2 inoculation [47]. Long term viremia or infection could render the infected animals more prone to secondary infection, which was also observed in PCV3 infection [11]. It should be noted that it is still possible that the presence of the PCV3 DNA might not accurately reflect virion production, as in the case of porcine circovirus-like viruses [42]. Duration of PCV3 infection, chronic PCV3 infection, and coinfection with other pathogens should be studied.

### 3.3. Antibody Responses following PCV3 Infection in the Experimental Infection Studies

Infection did not resolve in any of the inoculated pigs in Studies A–C. Therefore, it might be possible that the immune responses controlling PCV3 infection did not yet occur under those experimental settings; viral and antibody titers were not measured in Study D. Moreover, neutralizing antibody was not examined in Studies A–C. Although anti-PCV3 antibodies were measured, those were overall anti-PCV3 antibodies determined by ELISA methods (Table 1). 

The onset of antibody response seemed to be negatively correlated with PCV3 disease severity. In Study A (PDNS-like disease), anti-PCV3 antibody was first observed at 21 dpi (blood collection at 0, 7, 14, 21 and 28 dpi) which was in contrast with Studies B and C (subclinical infection) in which the anti-PCV3 antibody was readily found at 7 dpi. Mechanisms underlying the late antibody response in Study A are not known. More studies are needed to determine whether this might involve immune evasion in higher disease severity settings (such as in Study A). 

## 4. Possible Factors Contributing to the Varied Outcomes between the Experimental Infection Studies

Factors affecting PCV3 associated diseases are not yet known. However, since Studies A–D showed different disease outcomes, exploring the varied study parameters (Table 1) could be useful in identifying these factors. 

### 4.1. Virus Strain

Studies A (PDNS-like disease), B (subclinical infection), and D (diarrhea) used different strains of PCV3 for the inocula. At the genome level, nucleotide variation of these PCV3 strains can be found at various sites (data not shown). Deduced amino acid variations are shown in Table 4. However, influence of PCV3 genetics on the pathogenesis is currently unknown.

At least three open-reading-frames (ORFs) have been proposed for PCV3, including ORF1, ORF2, and ORF3. Based on other circoviruses, these ORFs encode the replicase protein (ORF1), the capsid protein (ORF2), and apoptosis-inducing protein (ORF3). The function of these ORFs of PCV3 should be further clarified. The start codons of PCV3 ORF1 and ORF3 have been proposed, but not yet confirmed [7]. The ORF3 start codon has been proposed to be TCG at nucleotide position 1900–1902 (ORF3_231_, 231 amino acid protein), or ATG at nucleotide position 62–64 (ORF3_177_, 177 amino acid protein) [7,48]. However, the TCG codon at position 1900–1902 (the start codon of ORF3_231_) is absent in all three PCV3 strains where TTT or TCT were found at this position instead.

In addition to the genetic difference of these viruses, the clinical diseases and outcomes observed in the farms of origin also varied (Table 1). These disease presentations were successfully reproduced in each study (PDNS-like disease in Study A, myocarditis in Study B, and diarrhea in Study D). It might not be unexpected that virus genetics could be one of the underlying causes of the discrepancies. Recently, a PCV3-infectious-clone construction method has been established [49]. Therefore, identification of genetic variation responsible for the varied clinical disease outcomes is possible. 

### 4.2. Virus Infectivity

Although it is likely that virus infectivity level can influence the disease outcome, it is not possible to compare the infectivity of the inocula in Studies A–D. Only Study A measured the infectivity of the inoculum (in TCID_50_ unit). The inocula of Studies B–D were measured as PCV3 genomic copies (gc). The gc unit only reflects the amount of virus, but cannot directly indicate the infectivity. PCV3 titers in the inocula of Studies B–D varied greatly, from approximately 10^6.5^ to 10^12^ gc. However, it is not known whether the inocula with higher gc would have higher infectivity. In addition, the natural PCV3 infection dose is not yet elucidated. The results from Study A might be partly due to higher virus titer in the inoculum. Further studies are needed to clarify the influence of PCV3 inoculation titers on the disease outcomes.

### 4.3. Coinfection

Only Studies A and B used pure PCV3 isolates as the inocula while in Studies C and D, tissues or content from PCV3-infected pigs were used. Contamination with other pathogens was possible and could result in coinfection. The influence of coinfection with other pathogen(s) on disease outcome has already been shown in PCV2 [50,51]. Although various swine pathogens were ruled out in these four studies, unspecified or unknown pathogens/agents in the inocula were still possible. PCV3 coinfection studies are needed.

### 4.4. Route of Infection

Intramuscular, intranasal, and oral inoculation routes were used in the studies. The role of inoculation route and its effect on PCV3 disease pathogenesis, and which route(s) most represent the field scenarios, are not yet known. 

Influence of inoculation route might rely on the primary replication sites of the virus, which is not known for PCV3. In Study C, histopathological changes started between 21–42 dpi and PCV3 replication was found in various tissues (Table 3) at 42 dpi (virus replication was not studied at 21 dpi). It is not known whether these tissues were the primary replication sites for PCV3. It might be possible that virus replication occurs in other tissues without obvious histopathological changes during the early phase of infection. Study C inoculated each pig via both intranasal and intramuscular routes. It should also be noted that the observed tissue tropism might be influenced by the inoculation route. It is still not known whether each route could affect the virus replication and pathogenesis differently. The influence of inoculation route on PCV3 infection kinetics/dynamics and disease outcome should also be studied.

### 4.5. Immune Stimulation

Comparison between pigs given PCV3 alone or with keyhole limpet hemocyanin (KLH) in incomplete Freund’s adjuvant (IFA) was conducted in Study A and C. In both studies, similar results between PCV3 infection alone and PCV3 infection with KLH in IFA were similar. Therefore, the influence of immune stimulation might be minimal, at least in these settings. 

### 4.6. Pig Breed 

Pig breeds were only provided in Study A. The impact of pig genetics on PCV3 associated diseases is unknown. It has been shown that the genetic background of pigs can affect the severity of PCV2-induced disease. Landrace pigs developed more severe lymphoid depletion after experimental PCV2 infection compared with Duroc, Large White pigs [52], and Pietrain pigs [53]. Yorkshire x Landrace pigs also showed more severe lung lesions after PCV2 infection compared with the Chinese indigenous breed called Laiwu [54]. The underlying mechanisms were not known. Association between pig genetics and PCV3 associated diseases should be further studied.

### 4.7. Microbial Colonization

Microbiota can affect the outcome of virus infection by various mechanisms. It is possible that the microbiota could also be one of the factors influencing PCV3 disease outcomes. Further studies are needed. 

Commensal microbiota can have a dual role in viral infection, either enhancing or suppressing the disease, and could be dependent on both the microbiota communities and the virus itself. The role of microbiota on virus infection has been previously reviewed [55,56,57]. Studies B and C (subclinical infection) used caesarian-derived/colostrum-deprived (CD/CD) pigs while Study A (PDNS-like disease) used conventional specific pathogen free pigs. CD/CD pigs have been used in studying the pathogenesis of various swine viral diseases, mainly to avoid the passive immunity from the dams and infection of other pathogens [58,59,60,61]. However, different microbial colonization can be observed between conventional and CD/CD pigs, such as in the respiratory and gastrointestinal tracts [62,63]. Whether the lower disease severity found in Studies B and C compared with Study A was due to the impact of using CD/CD pigs should be further clarified. Conversely, it is also possible that microbiota in Study A might enhance the disease severity when compared with Studies B and C. Several commensal bacterial species in the swine upper respiratory tracts can become opportunistic pathogens leading to respiratory diseases [64,65,66]. Involvement of bacteria in the development of lesions in the lungs of pigs in Study A was not identified. Although bacterial culture was not performed, neutrophil recruitment in the lung tissues was observed, which is suggestive of bacterial infection.

Notably, PCV3 infection in conventional pigs was found to alter the gut microbiota profile [43]. In the microbiota study, the PCV3 strain used was the same isolate as in Study A, which showed severe PDNS-like disease. Certain altered bacterial families following PCV3 infection have been suspected to play a role in other swine viral diseases as well. For example, PCV3-inoculated piglets showed significantly lower amounts of the *Ruminococcaceae* family [43]. It has been shown that this bacterial family is associated with reduced disease severity caused by various swine viruses, including PCV2, porcine reproductive and respiratory syndrome (PRRSV), and porcine epidemic diarrhea virus (PEDV) [67]. The role of microbiota in PCV3 infection is needed. 

Based on the recent experimental infection studies, several aspects of PCV3 pathogenesis began to become more apparent. However, many issues still await further investigation. It is now confirmed that PCV3 can induce pathological changes in the host. In some scenarios, it might only be observed at the microscopic level without obvious clinical signs (subclinical infection). On the other hand, severe clinical outcomes were induced in some settings. Lastly, viral and host factors could be extremely crucial and should not be ignored in future studies. 

## 5. Concluding Remarks

PCV3 is an emerging virus recently found in pigs showing various disease manifestations. However, knowledge on the pathogenesis of the diseases is limited. Hence, this review article provides a comprehensive understanding based on the experimental infection studies of the pathogenesis of PCV3-associated multi-organ inflammation. Regarding PCV3 pathogenicity, the virus itself has the potential to establish infection in inoculated pigs. However, infected pigs did not consistently develop clinical signs or lesions. Factors involving PCV3 disease development need to be clarified and investigated. It should be noted that primary PCV3 clinical outcome involves multi-organ inflammation. Three major mechanisms are proposed and might plausibly explain the pathogenesis of multi-organ injuries by PCV3 infection: (i) virus-induced injury (e.g., virus-induced apoptosis); (ii) immune-response-mediated mechanisms (e.g., immune system responding to eliminate infected cells, type IV hypersensitivity); and (iii) immune-complex-mediated mechanisms (type III hypersensitivity). However, many questions remain unanswered. Future directions for PCV3 research are needed to better understand and elucidate the precise pathogenesis. 

## Figures and Tables

**Table 1:** Selected parameters of study methods from experimental studies.

Parameters	Study A	Study B	Study C	Study D
Virus				
Name ^a^	LY (MF318451)	ISU27734 (MK058528)	N/A	JX (MK656956)
Origin	China, 2015	USA, 2018	N/A	China, 2018
Farms of origin				
Clinical signs/lesions	PDNS-like lesions	Lymphocytic myocarditis	N/A	Diarrhea
Affected pigs	Piglet	Piglet (8 days)	Fetus and suckling piglet	Suckling and weaned pigs
Inocula				
Infectious material	Virus isolate (infective clone)	Virus isolate	Tissue homogenate	Intestinal content
Route and titer ^b^	IN: 2 × 10^6.53^ TCID50	IN: 6.6 × 10^10^ gc IM: 6.6 × 10^10^ gc	IN: 2.04 × 10^11^ gc IM: 6.38 × 10^12^ gc (twice, 7 days apart)	Oral: 3.0 × 10^6.5^ gc
Inoculated pigs				
Breed	Duroc × Large White	N/A	N/A	N/A
Age	4 and 8 weeks old	6 weeks old	5 weeks old	3 weeks old
Farrowing status	Conventional	CD/CD	CD/CD	N/A
PCV3 detection				
Virus titer	qPCR (ORF2)	qPCR (ORF2)	qPCR (ORF2)	None
Tropism	IHC (PCV3 antigen)	ISH (ORF1 mRNA)	ISH (ORF1 mRNA)	None
Antibody detection				
Target	Capsid	Capsid	Capsid	None
Isotype	N/A	IgM and IgG	IgM and IgG	None
Duration of studies	28 days	28 days	42 days	7 days

Study A [18]; Study B [17]; Study C [19]; Study D [13]; N/A, data not available; gc, genomic copy; IN, intranasal; IM, intramuscular; IHC, immunohistochemistry; ISH, in situ hybridization; CD/CD, cesarian-derived and colostrum-deprived; TCID50, median tissue culture infectious dose; ORF, open reading frame. ^a^ GenBank accession number is in parenthesis. ^b^ Virus titers were shown as total titers (calculated from ‘volume x concentration’ reported in the original papers).

**Table 2:** Scores of each disease parameter from Studies A, B and C.

Parameter	Study A	Study B	Study C
Disease severity	+++	+	+
Number of affected tissues	+++	+	++
Degree of tissue tropism ^a^	+++	+	++
Peak viremia titer	+++	+	++
Onset of anti-PCV3 antibody ^b^	+++	−/+	+

Study A [18]; Study B [17]; Study C [19]; Score, ranged from + = lowest value, to +++ = highest value among the three studies. ^a^ Tropism was determined by immunohistochemistry targeting PCV3 antigen (Study A) or in situ ybridization targeting PCV3 ORF1 mRNA (Study B and C). ^b^ + = shortest duration, +++ = longest duration.

**Table 3:** Distribution of PCV3 (IHC or ISH) and microscopic lesions (H&E).

Organ	Study A	Study B	Study C
Heart	IHC: + Myocarditis Myolysis and epicardial necrosis Hemorrhage Eosinophil infiltration	ISH: + Lymphoplasmacytic myocarditis Periarteritis	ISH: + Lymphoplasmacytic myocarditis and periarteritis
Kidney	IHC: + Interstitial glomerulonephritis	ISH: + Lymphoplasmacytic interstitial nephritis Periarteritis	ISH: + Lymphoplasmacytic interstitial nephritis and periarteritis
Intestine	IHC: + Lymphocyte infiltration Epithelial degeneration/necrosis Eosinophil infiltration	ISH: − Lymphoplasmacytic periarteritis Arteritis of the serosa	ISH: + Lymphoplasmacytic periarteritis and arteritis of the serosa
Spleen	IHC: + Lymphoid depletion/necrosis Hemorrhage	Absent (ISH and H&E)	ISH: + Lymphoplasmacytic periarteritis and arteritis
Liver	IHC: + Congestion Hepatocyte atrophy	Absent (ISH and H&E)	ISH: + Lymphoplasmacytic hepatitis
Lung	IHC: + Lymphoplasmacytic and histiocytic bronchointersitial pneumonia	Absent (ISH and H&E)	Absent (ISH and H&E)
TLN	IHC: + Lymphocytic necrosis Congestion and hemorrhage Eosinophil infiltration	Absent (ISH and H&E)	Absent (ISH and H&E)
MLN	IHC: + Lymphoid depletion/necrosis Eosinophil infiltration	Absent (ISH and H&E)	Absent (ISH and H&E)
ILN	IHC: + Lymphoid depletion Hyperplasia of epithelial-like cells Eosinophil infiltration	Absent (ISH and H&E)	Absent (ISH and H&E)
Brain	N/A (IHC and H&E)	Absent (ISH and H&E)	ISH: − Lymphoplasmacytic encephalitis with perivasculitis

Study A [18]; Study B [17]; Study C [19]; Study D [13]; IHC, immunohistochemistry; ISH, in situ hybridization; H&E, hematoxylin and eosin staining (histopathology); +, present; -, absent; N/A, data not available; TLN, tracheobronchial lymph nodes; MLN, mesenteric lymph nodes; ILN, inguinal lymph nodes; Study D reported only intestinal lesions, in which, epithealial degeneration and necrosis was found.

**Table 4:** Amino acid sequence variation of PCV3 strains from Study A, B, and D.

Amino Acid Positions of Each ORF	Study A LY (MF318451 ^a^)	Study B ISU27734 (MK058528 ^a^)	Study D JX (MK656956 ^a^)
*ORF1*			
aa 122	S	S	A
aa 278	C	F	C
*ORF2*			
aa 24	V	A	V
aa 27	K	R	K
aa 95	F	S	S
aa 150	L	I	I
*ORF3_231_*			
aa 1	F	S	F
aa 4	D	G	D
aa 12	S	A	S
aa 32	V	L	L
aa 227 ^b^	V	V	G

Study A [18]; Study B [17]; Study D [13]; ORF, open reading frame; aa, amino acid. ^a^ GenBank accession number. ^b^ Amino acid position 173 of ORF3_177_
